# Take flight: predation risk induces wing polyphenism and dispersal in the aphid *Myzus persicae*

**DOI:** 10.1002/ps.71141

**Published:** 2026-07-21

**Authors:** Jamin Ali, Adil Tonğa, Bashiru Adams, Geoff M Gurr, Rizhao Chen, Qiyun Li

**Affiliations:** ^1^ College of Plant Protection, Jilin Agricultural University Changchun China; ^2^ Sanjiang Laboratory Changchun China; ^3^ Entomology Department, Diyarbakır Plant Protection Research Institute Diyarbakir Turkey; ^4^ Delta Research and Extension Center, Mississippi State University Stoneville MS USA; ^5^ Gulbali Institute, Charles Sturt University Orange NSW Australia

**Keywords:** Aphididae, Coccinellidae, predation risk, *Myzus persicae* wing induction, predator‐induced avoidance

## Abstract

**BACKGROUND:**

Predation risk can generate strong non‐consumptive effects that influence prey physiology, morphology and behaviour, often leading to adaptive changes without direct consumption. In aphids, such effects may induce winged (alate) morphs that facilitate dispersal, yet the extent to which predation risk couples morphological plasticity with movement behaviour and survival advantages remains poorly understood, particularly under varying predator densities.

**RESULTS:**

We investigated how predation risk from the ladybird *Harmonia axyridis* influences wing induction, dispersal behaviour and survival in the aphid *Myzus persicae* using experimental series initiated with 50 nymphs or 50 adults. Predator presence above a certain density significantly increased the number of alate progenies in both nymph‐ and adult‐initiated series, with stronger effects at higher predator densities. In the adult‐exposure series, alate induction exhibited a non‐linear pattern, with significant increases in alate numbers at predator densities of five and ten, but not at one. In the nymph‐initiated series, a significant increase was observed only at the highest predator density (ten). Dispersal responses were morph‐specific: alate aphids showed significantly increased movement under elevated predation risk, whereas apterous aphids exhibited no significant change. Predation assays further revealed significantly lower consumption of alate compared with apterous aphids for both male and female predators, indicating a survival advantage associated with wing induction.

**CONCLUSION:**

These findings suggest that predator‐induced phenotypic plasticity integrates morphological and behavioural responses to enhance prey survival. This creates a potential trade‐off for biological control, because high predator densities may trigger winged morph production and facilitate pest dispersal to neighbouring crops; however, this assertion requires field validation. © 2026 The Author(s). *Pest Management Science* published by John Wiley & Sons Ltd on behalf of Society of Chemical Industry.

## Graphical abstract

Predator risk above a critical density induces winged morphs in *Myzus persicae*, promoting dispersal and reducing predation compared with wingless aphids. This density‐dependent phenotypic plasticity enhances survival but may compromise biological control by facilitating aphid dispersal.

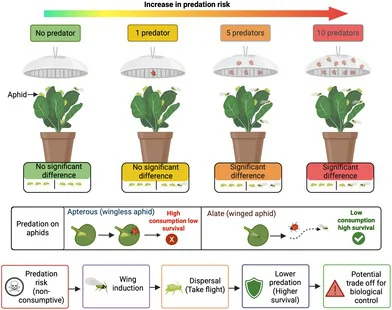

## 1 INTRODUCTION

Biological control is a key component of integrated pest management (IPM), in which natural enemies regulate pest populations and influence ecological dynamics.^1^, ^2^ Predation risk plays a crucial role in shaping the behaviour, development and life‐history strategies of herbivorous insects. Under predation risk, insects must balance the energy invested in growth and reproduction with the need to express traits or behaviours that enhance survival.^3^ This resource allocation trade‐off often leads to physiological changes, such as the development of traits or the adoption of escape behaviours, which help insects reduce vulnerability to predators.^4^ Such adaptive strategies, although beneficial for survival, may come at the cost of other life‐history traits, such as fecundity, as energy may be diverted from reproduction towards survival mechanisms.^5^ Understanding how predation influences these adaptive strategies is essential for refining pest management approaches, because it highlights the complex dynamics of predator–prey interactions, including both consumptive (direct predation) and non‐consumptive (indirect effects such as altered behaviour or physiology) impacts.^6^

The peach‐potato aphid, *Myzus persicae* (Sulzer) (Hemiptera: Aphididae), is a global agricultural pest because of its broad host range (feeding on over 500 plant species across more than 40 plant families) and its capacity to evolve resistance to insecticides.^7^, ^8^ Hosts include a diverse array of crops, with major agricultural staples such as brassicas and potatoes being particularly vulnerable. *Myzus persicae* causes both direct and indirect damage to crops.^9^, ^10^ Direct damage occurs through feeding, where aphids extract plant sap, weakening plants and stunting their growth. Indirectly, aphids contribute to the spread of plant pathogenic viruses, which can lead to widespread crop losses.^11^ The aphid's phenotypic plasticity in response to environmental stressors further complicates pest control efforts. It exhibits different morphs, including alate and apterous forms, the proportion of which in a given population is influenced by various biotic and abiotic factors.^12^, ^13^, ^14^, ^15^ Critically, alate induction is a maternally controlled process spanning generations, in which tactile stimulation (crowding) and host plant quality serve as primary cues.^16^, ^17^ This prenatal and/or postnatal maternal programming^17^ enables aphids to produce alate offspring in anticipation of deteriorating conditions. Although day length and temperature modulate responsiveness, predator cues likely act synergistically with these core stressors to trigger alate production. The alate morphs serve an important role in dispersal, allowing aphids to move to distant plants or new habitats when survival pressures or resource competition are high, thereby avoiding localized competition and enhancing their chances of survival.^18^, ^19^ Further, alate morphs are critical for the epidemiology of viruses, because they can infest distant crop areas, facilitating the movement of plant pathogens across fields.^20^, ^21^

In the context of predation risk, the distinction between alate and apterous morphs in *M. persicae* is of particular interest. The dispersal ability of the alate forms not only helps aphids avoid high predation pressure, but also plays a role in the movement of aphids to new crops. By contrast, apterous aphids are typically more sedentary and, although they may exploit a host plant for longer, they are more vulnerable to predators. The development of alate morphs as an escape strategy is a clear example of how aphids balance between resource allocation for growth and the need to invest in survival mechanisms.^22^ This morphological plasticity raises important questions about aphids’ adaptive strategies under natural enemy pressure and their potential for pest management.

Although the consumptive effects of natural enemies on herbivorous insects have been extensively studied, research on non‐consumptive effects, particularly those involving changes in prey development and behaviour due to predator cues, remains underexplored.^5^, ^23^, ^24^, ^25^ This study addresses this gap by investigating how predator‐associated cues from *Harmonia axyridis* (Pallas) (Coleoptera: Coccinellidae) influence alate morph induction in *M. persicae* and whether alate and apterous morphs differ in predation susceptibility. Although the consumption rate of *M. persicae* by *H. axyridis* has been documented previously, whether the two morphs differ in predation susceptibility remains unknown; comparing them is essential to evaluate the adaptive benefit of predator‐induced wing formation. We hypothesized that exposure to predator cues triggers alate morph development, offering an escape strategy from predation. The development of wings may make aphids less accessible to predators, potentially reducing predation rates and affecting predator–prey dynamics.^26^, ^27^ Understanding these non‐consumptive effects is crucial for enhancing IPM strategies, because it provides deeper insight into the ecological interactions between aphids and their natural enemies, and how these interactions can be harnessed to optimize biological control in agricultural settings.^28^, ^29^

## 2 MATERIALS AND METHODS

### 2.1 Insects

*Myzus persicae* founders were purchased from Qu Shou Natural (Nanyang, China), and a laboratory colony was established on field mustard (*Brassica rapa*) plants in rearing cages (60 × 60 × 60 cm) under controlled environmental conditions [22 ± 2 °C, 45 ± 5% relative humidity (RH), 16:8 h light dark (L/D) photoperiod]. We acknowledge that this approach may introduce genetic variation, which could limit the generality of our findings to specific genotypes. To establish the colony, adult aphids from the commercial stock were placed on a clean *B. rapa* plant for 24 h to produce nymphs. The adults were then removed, and the nymphs produced were reared to establish the colony. All experiments used F1 or F2 progeny from this colony. One‐day‐old nymphs and newly developed adult apterous aphids from this colony were used in all experiments. The source colony was maintained at low density on healthy 4‐week‐old *B. rapa* plants to minimize baseline alate production from crowding or poor nutrition. Only apterous adults were used to produce nymphs for experiments, and all 1‐day‐old nymphs were collected from the same cohort to ensure random distribution of any maternal effects across treatments. *Harmonia axyridis* founders were purchased from the Keyun, Henan, China Company, and they were reared on *M. persicae* at 22 ± 2 °C, 45 ± 5% RH and 16:8 h L/D photoperiod. All experimental predators were adults of similar age to minimize age‐related variation in behaviour and cue production.

### 2.2 Plants

*Brassica rapa* plants were grown from seed (Hebei Dayu Seeds, Cangzhou City, Hebei, China) at 22 ± 2 °C, 45 ± 5% RH, 16:8 h L/D photoperiod in 7‐cm‐diameter pots filled with sterilized potting mix (Jiangsu Siweibo Biotechnology Co., Ltd, Xuzhou City, China). All experiments were conducted using 4‐week‐old plants (Biologische Bundesanstalt, Bundessortenamt und Chemische Industrie growth stage 14).^30^

### 2.3 Induction of alate morphs in the presence of predators

To study the effect of predator presence on the development of alate morphs in *M. persicae*, a plastic bowl (12 cm in diameter, 8 cm in depth and 7 cm at the narrow end) was used (Fig. 1). The opening of the bowl was covered with insect netting to allow airflow while confining predators. A rope was attached to the bottom of the bowl and connected to the upper part of an insect cage (60 × 60 × 60 cm), with the bowl hanging upside‐down in the cage. A hole near the rope attachment allowed predators to be released into the bowl, after which it was plugged with cotton wool to prevent their escape. This experiment used a single plant per cage and consisted of four predator density treatments: P0 (zero predators; control), P1 (one predator), P5 (five predators) and P10 (ten predators).

**Figure 1:**
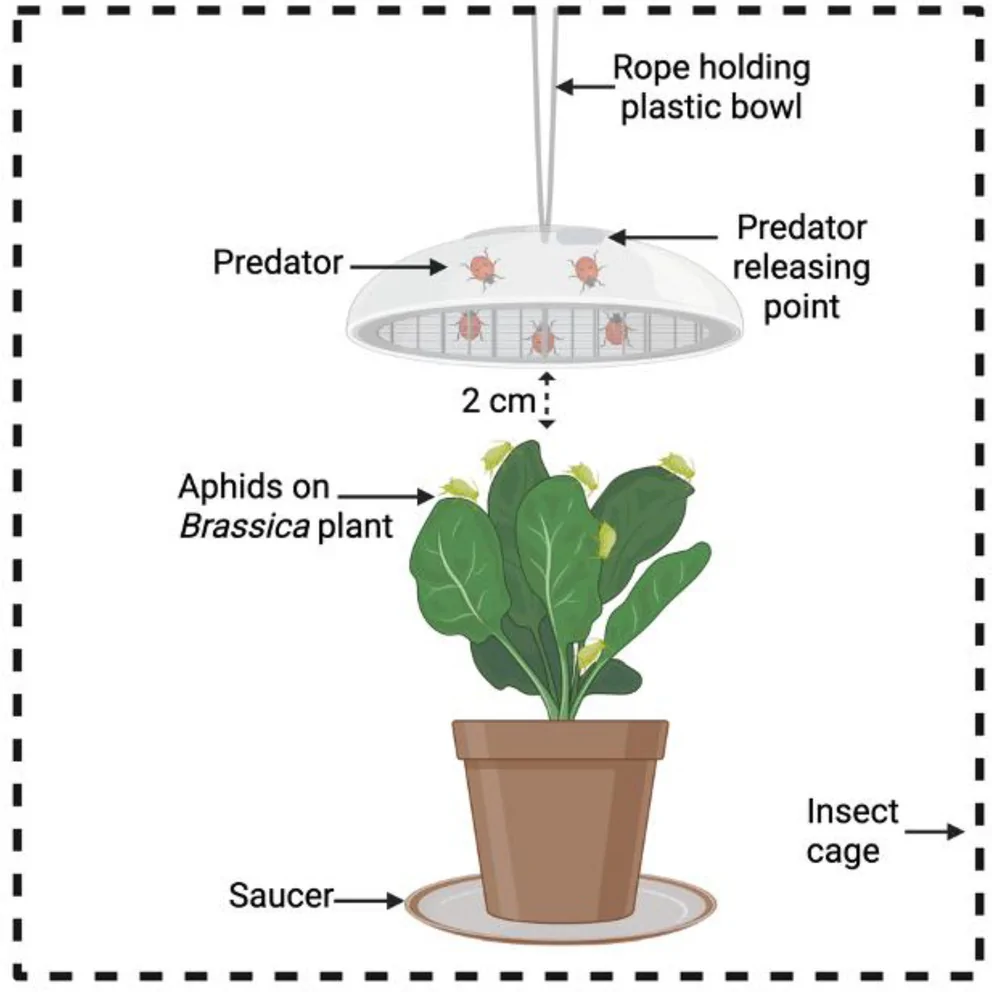
Experimental setup for assessing predator‐induced effects on alate morph development in *Myzus persicae*. Predators were housed inside a plastic bowl suspended 2 cm above the aphid‐infested *Brassica* plant, allowing exposure to predator‐associated cues without direct contact.

The experiment was conducted in two series: in series 1, the plant was inoculated with 50 one‐day‐old *M. persicae* nymphs to test for predator‐induced wing development in the progeny generation. In this series, any maternal programming for alate production would be equally present across all treatments at the outset; thus, differences in alate numbers among predator density treatments can be attributed to the non‐consumptive effects of predator exposure. The alate aphids counted over the 14‐day period represent the F1 progeny of the original 50 nymphs, consistent with the maternal programming mechanism of wing induction. In series 2, plants were inoculated with 50 adult aphids. In both series, a water‐filled saucer was placed beneath the plant pot for irrigation and to prevent aphids from escaping by walking. This setup ensured that aphids could not directly enter the predator containment bowl (thus avoiding physical contact), while maximizing exposure to predator‐associated cues between the aphids on the plant and the predators inside the bowl. Predators were replaced daily with new individuals that had not been used in previous experiments and were starved for 2 h before use to standardize hunger level. The number of alate aphids in each cage was recorded daily for 14 days. After each daily recording, the alate aphids were removed from the cage to prevent recounting and to maintain accurate daily counts. The experiment was conducted in a greenhouse under controlled environmental conditions (22 ± 5 °C, 45 ± 5% RH, 16:8 h L/D photoperiod). For each treatment, half of the replicates involved male predators and the other half female predators, to avoid any sex‐specific bias in predator impact. Predator sex was balanced but was not included as a factor in the statistical models, because the experiment was not designed to test sex‐specific effects. The balanced design ensures that any sex‐specific effects would be equally distributed across treatments. During daily counts, the entire inner surface of each cage, the water‐filled saucer, and the walls of the inverted predator bowl were inspected for any aphids that may have dispersed from the host plant. No apterous aphids were observed on these surfaces throughout the 14‐day experimental period, indicating that long‐distance walking dispersal from the host plant did not occur. However, we cannot exclude the possibility of short‐term within‐plant movement or temporary dropping during the observation period. Each group was replicated ten times.

### 2.4 Movement of apterous aphids in the presence of predators

To evaluate the effect of predation risk on aphid dispersal, we used a setup based on the experiment for the induction of alate morphs (see Section 2.3), with the following modifications. A plant inoculated with 50 adult apterous aphids was placed in the centre of the cage (60 × 60 × 60 cm) (shown in Fig. 1), with four uninfested plants positioned in the corners. The saucer beneath the central plant was left dry (without water) to allow aphid movement. After 24 h, the number of aphids that had moved to the corner plants (and other surfaces in the cage, including walls and floor) was recorded. The same predator density treatments (zero, one, five and ten predators) were applied. Each treatment was replicated ten times. The suspended predator bowl was hung in the centre of the cage at the top of the plants (2 cm above the central plant), identically positioned for both apterous and alate dispersal assays. As in the induction experiment, equal numbers of male and female predators were used across replicates, and predators were starved for 2 h prior to the assay.

### 2.5 Movement of alate aphids in the presence of predators

This experiment followed the identical procedure described for apterous aphid dispersal above, but using alate aphids. All other conditions, including predator density treatments (zero, one, five and ten predators), remained the same. Each treatment was replicated ten times. The same bowl position (centre of the cage, 2 cm above the central plant) was used as described in Section 2.4, with equal numbers of starved (2 h) male and female predators.

### 2.6 Consumption of alate and apterous aphids

To determine whether alate and apterous morphs of *M. persicae* differ in their susceptibility to predation, and whether predator sex influences this pattern, we conducted consumption experiments with adult *H. axyridis*, using females in one experiment and males in another.

Before the experiments, adult beetles were starved for 2 h to standardize the hunger level. For each replicate, a potted plant infested with 100 aphids of same morph and life stage (adult) was placed inside a 40 × 40 × 40 cm cage. Cages were securely sealed during the 24‐h assay to prevent aphid escape. One set of cages contained only apterous morphs (100 individuals per cage), and the other set contained only alate morphs (100 individuals per cage). All aphids were weighed on an electronic microbalance (Sartorius Scientific Instruments (Beijing) Company Ltd, Beijing, China) before placing them on plants. Biomass was measured as total group weight of 100 aphids per cage, rather than individually.

A single randomly selected adult predator of the respective sex was introduced onto the plant and allowed to forage for 24 h, after which time the number of unconsumed aphids was counted as a function of unconsumed aphid rate (%) and the surviving aphids reweighed. Aphids that were not found alive were considered consumed. The entire cage interior (plant, pot, cage walls and floor) was thoroughly inspected for aphid carcasses or live aphids at the end of the assay. Biomass consumption was calculated based on the difference in aphid weight before and after (for unconsumed aphids) the predation period. Preliminary control tests conducted without predators confirmed that natural aphid mortality was negligible over 24 h.

We acknowledge that the natural biomass gain of surviving aphids was not quantified; therefore, biomass consumption estimates are presented as uncorrected values and should be interpreted with this limitation. Our primary consumption metric is the number of unconsumed aphids (count data), which is unaffected by biomass gain. Predator sex was balanced, and predators were starved for 2 h prior to the assay, following the same protocol as Sections 2.3 and 2.4. Fifteen replicates were conducted for each morph and predator–sex combination. This design allowed assessment of both the number and biomass of consumed aphids, as well as the comparison of predation effects between male and female *H. axyridis* on alatae and apterous aphids.

### 2.7 Statistical analyses

We analysed longitudinal alate aphid count data over time using zero‐inflated negative binomial generalized linear mixed‐effects models (GLMMs) to account for overdispersion, excess zeros, and repeated measurements within plants using the package glmmTMB. Two different model structures were selected for the number of alate aphids produced upon the release of nymphs and adults. The first included treatment, day and their interaction as fixed effects, with plant as a random intercept to account for repeated measures, and treatment and day in the zero‐inflation component for nymph‐initiated series. The second model used the same structure but replaced the linear day term with a B‐spline smoothing term from the package splines to capture potential non‐linear trends over time for adult‐initiated series. Model fit was guided by visual inspection of residual diagnostic plots, including QQ plots, and by the stability of parameter estimates.

Significance of fixed effects was assessed using type II Wald chi‐squared tests implemented in the car package's Anova() function. The count data regarding the total number of alate aphids produced on each plant was tested using a generalized linear model (GLM) with Poisson distribution. The rate of apterous and alate aphids not consumed by predators and the rate of those moved were modelled with GLMs using quasibinomial distribution. The weight of remaining aphids was compared with initial aphid release using a gaussian distributed GLM model. The Anova() function from the car package generated likelihood ratio tests results for GLMs. Pairwise comparisons among treatment levels were conducted using Tukey‐adjusted simultaneous tests implemented with the glht function in the multcomp package. All contrasts were evaluated on the linear predictor scale of the respective models. All figures were generated using the package ggplot2, with the help of tidyr, ggdist, officer and rvg. All analyses were conducted in R (v.4.5.0; R Development Core Team, 2024).

## 3 RESULTS

### 3.1 Induction of alate morphs in the presence of predator

The number of alate *M. persicae* was significantly affected by predation risk (treatment_nymph_; *χ* ^2^_df=3_ = 9.74, *P* = 0.021 and treatment_adult_; *χ* ^2^_df=3_ = 59.27, *P* < 0.001) and time factor (treatment_nymph_; *χ* ^2^_df=1_ = 75.83, *P* < 0.001 and treatment_adult_; *χ* ^2^_df=5_ = 283.24, *P* < 0.001) with divergence in the effect of treatment × time interaction (treatment × time_nymph_; *χ* ^2^_df=3_ = 6.43, *P* = 0.092 and treatment × time_adult_; *χ* ^2^_df=15_ = 26.30, *P* < 0.001). Consistently, predation risk treatment also significantly affected total number of alate aphids produced over the entire experiment, both when starting with nymphs (treatment_nymph_; *χ* ^2^_df=3_ = 63.18, *P* < 0.001) and adults (treatment_adult_; *χ* ^2^_df=3_ = 246.12, *P* < 0.001). Specifically, alate production was highest in the P10 treatment, and the P10 and P5 treatments were significantly higher than the P1 and control treatments (Fig. 2). In nymphs, P10 and P5 were significantly different from each other (Fig. 2(B)), but no significant difference was observed between P10 and P5 in adults (Fig. 2(D)).

**Figure 2:**
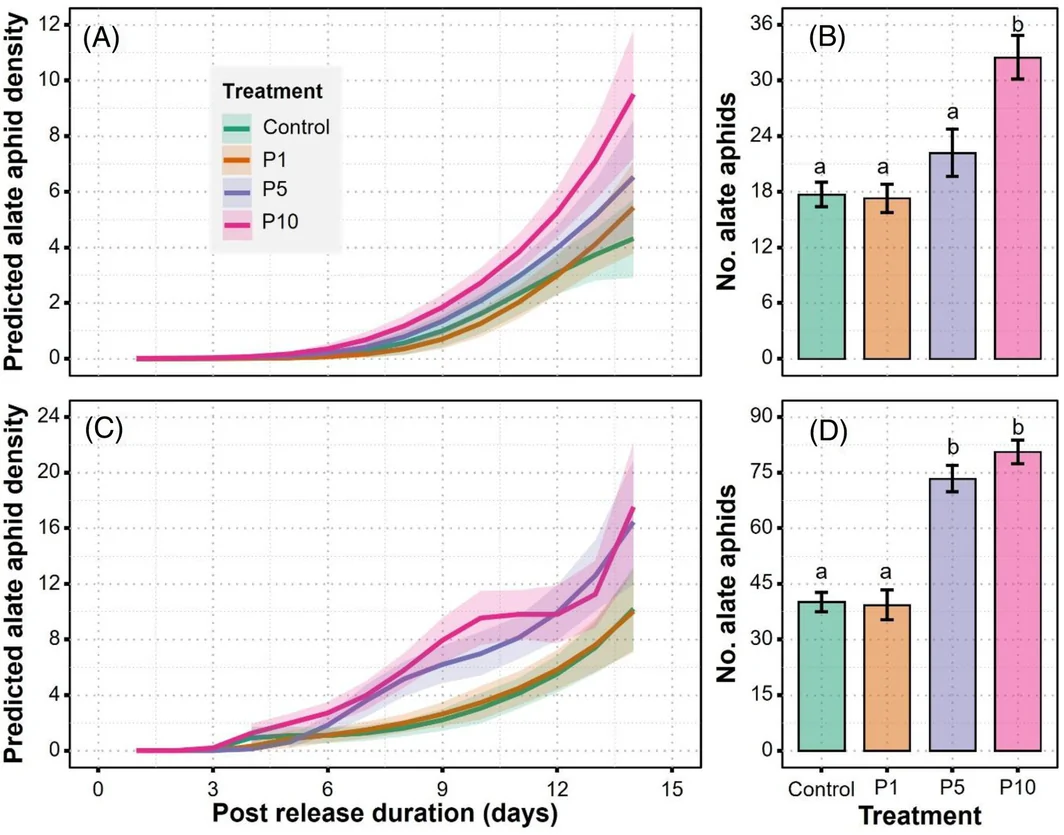
Model‐predicted number of alate *Myzus persicae* occurred over time (curves equipped with 1.96× SE intervals) upon nymph (A) and adult (C) release by generalized linear mixed models (GLMMs, see Section 2.7 for details). The average (±SE) of total number of alate *M. persicae* produced under treatments with different numbers of predators, *Harmonia axyridis* (P1 = single predator, P5 = five predators, P10 = ten predators, control = no predators) upon nymph (B) and adult (D) releases. Different lowercase letters indicate significant differences between treatments based on Tukey's post‐hoc comparison tests at the level of *P* < 0.05 following respective models.

### 3.2 Movement of apterae and alatae in the presence of predators

The movement rate of apterous *M. persicae* did not change as predation risk increased (*χ* ^2^_df=3_ = 1.09, *P* = 0.779) (Fig. 3 (A)), but the movement of alatae did (*χ* ^2^_df=3_ = 11.35, *P* < 0.01) (Fig. 3 (B)). The number of alate aphids showing movement was significantly higher in treatments with ten predators than in the control, whereas those exposed to a single predator and five predators did not differ from controls.

**Figure 3:**
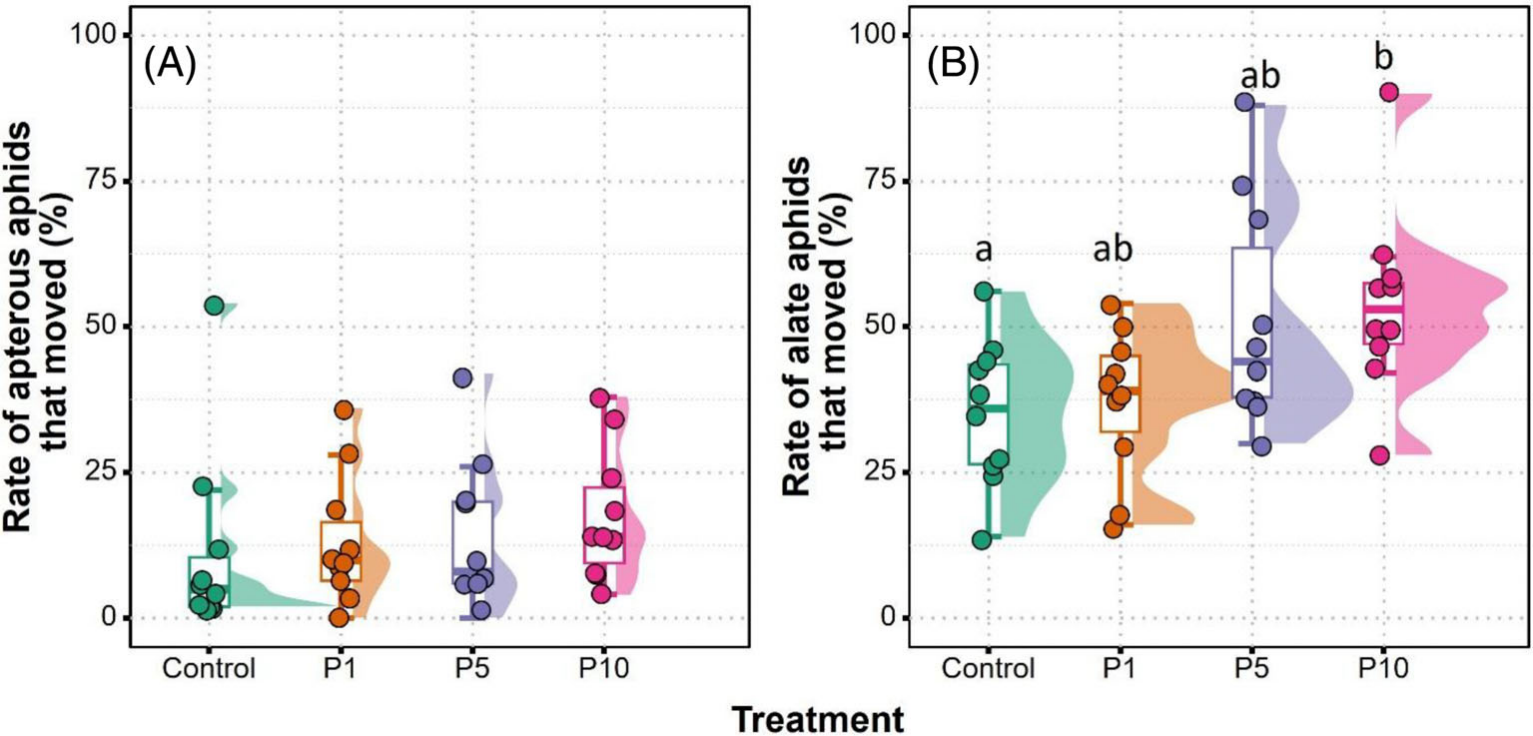
Number of apterous (A) and alate (B) *Myzus persicae* (depicted as boxplots accompanied by violins) under treatments with different numbers of predators, *Harmonia axyridis* (P1 = single predator, P5 = five predators, P10 = ten predators, control = no predators). Different lowercase letters indicate significant differences between the number of alate aphids that moved under different predation risk treatments based on Tukey's post‐hoc comparison test at the level of *P* < 0.05 following respective models (GLMs, see Section 2.7 for details).

### 3.3 Consumption of alate and apterous aphids

The number of *M. persicae* consumed by female (*χ* ^2^_df=1_ = 122.61, *P* < 0.001; Fig. 4(A)) and male (*χ* ^2^_df=1_ = 117.09, *P* < 0.001; Fig. 4(B)) *H. axyridis* differed significantly between alatae and apterae. Alate aphids exhibited a significantly higher survival rate than apterous aphids (*P* < 0.001). Count‐based consumption (number of surviving aphids) served as our primary metric, because it is unaffected by the natural biomass gain of survivors over the 24‐h assay period. Accordingly, the weight of both apterous and alate aphids remaining on plants was significantly lower compared with pre‐treatment when aphids were exposed to either female (apterous: *χ* ^2^_df=1_ = 2946.7, *P* < 0.001; alate: *χ* ^2^_df=1_ = 894.46, *P* < 0.001) or male (apterous: *χ* ^2^_df=1_ = 1078.5, *P* < 0.001; alate: *χ* ^2^_df=1_ = 1442.5, *P* < 0.001) predators (Supporting Information, Fig. S1). The biomass data (Supporting Information, Fig. S1) are presented as uncorrected values and are supplementary; natural weight gain of surviving aphids was not quantified.

**Figure 4:**
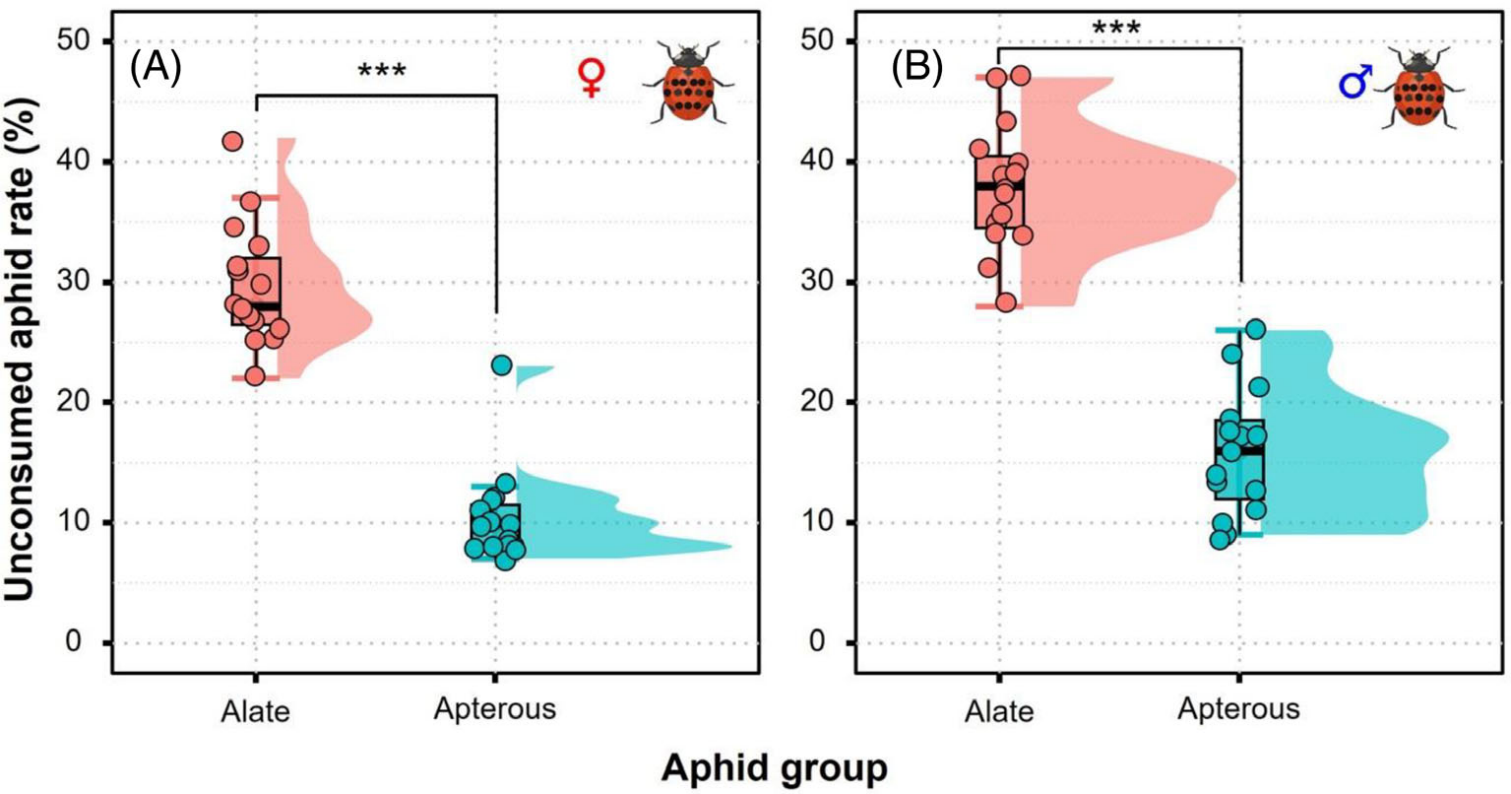
The rate (%) of unconsumed *Myzus persicae* (depicted as boxplots accompanied by violins) in alate and apterous groups following predation by a single female (A) and male (B) *Harmonia axyridis*. Data represent per cent live aphids remaining after 24 h, which are unaffected by natural biomass gain of survivors. Alate aphids exhibited a significantly higher survival rate compared with apterous aphids (***P < 0.001).

## 4 DISCUSSION

This study supports our hypotheses that predator cues induce wing formation in *M. persicae* as an escape strategy since alatae experience less predation relative to apterae. Our findings demonstrate: (1) predator presence significantly increased alate production in a predator density‐dependent manner, although the pattern differed between exposure series. In adults, a non‐linear response pattern was observed, with significant induction at five and ten predators but not at one predator. In nymphs, only the highest density (ten predators) triggered a significant increase over controls; (2) alate aphids exhibited predator density‐dependent dispersal (significantly higher movement at P5/P10); and (3) both predator sexes consumed significantly more apterous than alate aphids, confirming the survival advantage of winged morphs. These adaptive responses of the aphid to *H. axyridis* exposure highlight how non‐consumptive effects shape prey polyphenism and behaviour. The density‐dependent alate induction, where significant wing formation occurred at ten predators in the nymph series and at both five and ten predators in the adult series, reveals that the response pattern may vary with aphid life stage. This pattern indicates predator cues trigger wing development only above a critical cue intensity level, rather than through simple linear density dependency. The 14‐day experimental period captured maternal programming dynamics consistent with known stress‐response mechanisms,^16^, ^17^ where mothers exposed to predator threats produce winged offspring. In the nymph‐initiated series, the alates counted were the F1 offspring of the original nymphs, consistent with a transgenerational response (maternal programming) rather than a within‐generation effect. Moreover, the similar response magnitude at P5 and P10 suggests predator cues saturate the induction mechanism beyond a critical density.

Evidence of density‐dependent alate production supports *M. persicae*'s capacity for adaptive phenotypic plasticity,^31^, ^32^ extending similar enemy‐induced wing formation documented in other aphid species when exposed to predators and parasitoids.^33^ Notably, the non‐linear pattern was clearer in adult‐exposed aphids, where five predators were sufficient to trigger induction, whereas in nymph‐exposed aphids, only ten predators elicited a significant response. This suggests that sensitivity to predator cues may be influenced by developmental stage. The response aligns with tactile stress pathways described by Dixon,^17^ where mechanical stimulation from crowding induces wing development, here potentially simulated through predator movement cues. The investment in dispersal‐capable morphs represents a strategic resource reallocation, prioritizing escape over reproduction when predation risk exceeds critical thresholds. This response also represents adaptive polyphenism where aphids alter development in response to environmental cues.^34^ The development of alate morphs involves strategic resource allocation, with aphids investing in dispersal‐capable individuals that can escape high‐risk environments, potentially at the expense of other life‐history traits.^35^ Such shifts from growth/reproduction towards dispersal mechanisms are characteristic of insects facing predation pressure.^3^ For instance, predation risk reduced feeding and oviposition in Colorado potato beetle (*Leptinotarsa decemlineata* (Say)),^36^ while tephritid fruit fly (*Bactrocera dorsalis* (Hendel)) females exposed to mantids or their odours altered development and fecundity.^5^ Collectively, these findings show that prey adjust resource allocation under predation risk, prioritizing survival over reproduction.

Dispersal responses were morph‐specific, further validating our hypotheses. Alate aphids showed significantly increased movement at higher predator densities (P5/P10), consistent with an escape response leveraging their flight capability. By contrast, apterous aphids displayed minimal changes in dispersal to new plants, probably constrained by limited mobility and stronger host dependency.^26^, ^37^ However, predator presence altered their distribution on the original plant, because aphids often drop from the plant when threatened by actively searching predators and then climb back, settling in protected microhabitats such as leaf whorls, buds or lower leaf surfaces.^38^, ^39^ Such dropping behaviour is a well‐documented immediate escape tactic for wingless aphids,^40^ and studies confirm that actively searching predators like *H. axyridis* elicit significantly more dropping than furtive predators.^41^ Although our assays did not directly quantify these behaviours, they represent plausible local escape responses for apterous aphids. We cannot exclude the possibility that the cage size and assay duration may have limited our ability to detect treatment effects on apterous dispersal. Thus, although apterous aphids lack long‐distance dispersal options, they use active local escape behaviours (like dropping and microhabitat adjustment) in response to perceived predation risk. By contrast, alate morphs leverage flight for long‐distance escape and colonization of predator‐free plants. These findings underscore divergent predator‐avoidance strategies: alatae rely on longer‐distance dispersal, whereas apterous morphs depend on immediate behavioural escapes and local redistribution, albeit without the same potential for generational escape.

Predation bioassays suggest that alate morphs have a survival advantage, because both male and female *H. axyridis* consumed significantly more apterous than alate aphids. This difference could reflect multiple, non‐mutually exclusive mechanisms. Alate aphids may escape via flight, whereas apterous morphs lack this capability.^27^, ^42^ Alternatively, alates may be less detectable or accessible on the plant, differ in handling time or ease of capture, vary in nutritional quality or palatability affecting predator preference, or occupy different locations on the plant. Although flight ability is a plausible contributor, our separate‐arena design does not allow us to distinguish among these mechanisms. The observed outcome reflects a fundamental life‐history trade‐off: under predation threat, mothers can shift investment from producing many locally breeding, vulnerable apterous offspring towards producing fewer, dispersal‐capable alate offspring.^17^ Alate aphids can escape via flight to potentially safer habitats, aligning with the concept of enemy‐free space.^31^, ^43^, ^44^

Wing development diverts substantial resources from reproduction, creating a well‐documented fitness trade‐off between dispersal and fecundity.^26^, ^45^, ^46^ Our findings, together with prior work showing increased alate production in aphids under ladybird threat underscore this adaptive strategy.^43^ Although wings do not confer direct physical defence, they enable escape from high‐risk environments, representing a strategic allocation in which survival and colonization potential are prioritized at the cost of immediate local reproductive output.

Our findings show that alates of *M. persicae* are less susceptible to predation than apterous morphs. This difference may be due in part to flight capability, which apterous morphs lack,^47^, ^48^ but could also reflect differences in detectability, handling time, location on the plant, nutritional quality, palatability or predator preference. Consequently, although efficient reproducers, apterous aphids face a higher realized predation risk.^34^, ^43^, ^48^, ^49^ The induction of alates under predation risk thus represents a key adaptive strategy that reduces local predation pressure but complicates biological control. Although predators suppress local populations by consuming primarily apterous forms, their presence may inadvertently trigger alate production, enabling dispersal and spread.^31^, ^38^, ^40^ This dual role of predation risk, reducing immediate survival of apterous forms while promoting the dispersal of alates, has important implications for pest management.

A key caveat must be acknowledged when extrapolating our findings to field conditions. Alate induction is a transgenerational process requiring several days from maternal cue perception to offspring wing development.^16^, ^17^ Under naturally high predator densities in the field, consumptive mortality would likely eliminate aphids before alate offspring could complete development. The massive alate production observed in our study was possible because the inverted‐bowl setup prevented direct predation, isolating non‐consumptive effects. Therefore, rather than warning against high‐density spillover, our findings provide a mechanistic argument for maintaining stable, low‐level natural enemy populations. Low predator densities can exert sufficient consumptive pressure to suppress aphid populations without crossing the stress level that triggers mass wing induction. This demonstrates that biological control is not simply a ‘more is better’ scenario; optimal predator densities exist that balance local suppression with minimal risk of inducing dispersal morphs. However, these findings are from controlled cage experiments; field validation is needed to confirm whether similar patterns operate under natural conditions where aphids and predators interact more dynamically.

Based on our findings, we hypothesize that IPM strategies may need to account for the adaptive responses of pests to predation risk. However, field studies are required to test this hypothesis. We suggest that habitat management designed to support a robust yet balanced community of natural enemies may help maintain effective suppression without creating cue concentrations that could potentially trigger mass alate production. Furthermore, integrating biological control with complementary tactics, such as crop rotation, resistant cultivars and other cultural practices, can build ecosystem resilience and mitigate risks associated with predator‐induced dispersal. Ultimately, this study highlights the complex interplay between predation risk, pest behaviour and biological control efficacy, emphasizing the need for holistic management practices that enhance crop protection while minimizing unintended consequences.

We acknowledge methodological limitations in this study. First, in the consumption assay, although our count‐based data (number of surviving aphids) are robust and unaffected by aphid growth, our biomass‐based estimates do not account for natural weight gain of surviving aphids over the 24‐h experimental period. Consequently, the absolute biomass consumption values presented in Supporting Information, Fig. S1 are uncorrected and should be interpreted cautiously. However, the qualitative pattern of higher consumption of apterous aphids compared with alates is consistent across both count‐based and biomass‐based metrics, supporting our main conclusion that alate morphs experience a survival advantage under predation risk. Second, the predator containment bowl used in the induction experiment prevented direct physical contact but did not allow us to isolate which specific cue type (visual, chemical or vibratory) triggered alate production. Previous studies suggest that aphid alarm pheromone (such as (*E*)‐β‐farnesene) or predator‐derived volatiles may play a role, but their specific contributions in this system remain unknown. Future studies using controlled cue presentations (e.g. predator odour alone *versus* visual cues alone) would help elucidate the mechanistic basis of the observed responses. We also did not quantify total aphid abundance, because our focus was specifically on alate induction; therefore, we cannot exclude the possibility that differences in colony growth may have influenced raw alate counts. All treatments were identical except predator presence, so raw alate counts still reflect treatment effects; however, we cannot distinguish between morph induction and population growth effects. Third, although our separate‐arena consumption assay is appropriate for testing differential survival, it does not isolate whether the observed difference is due to escape ability, nutritional quality or predator preference. Future choice tests would help resolve this. Furthermore, future studies could specifically examine whether predator sex influences alate induction and dispersal, as this remains unexplored. Despite these limitations, our findings support that predator‐associated cues induce alate formation in *M. persicae*, conferring a survival advantage and highlighting the need to consider non‐consumptive effects in biological control.

## FUNDING INFORMATION

This work was supported by National Key Support Project for Foreign Experts (Northeast Special Program), ‘Development and Application of High‐Efficiency Lure Cores for Physiological Population of Asian Corn Borer in Northeast China’ (Project No. D20250105). Ministry of Science and Technology of China (Grant No. 2022YFD1500701) and the Sanjiang Laboratory Science and Technology Innovation Project (Grant No. SJ2025008) for supporting this work.

## CONFLICT OF INTEREST

The authors declare that they have no conflict of interest.

## AUTHOR CONTRIBUTIONS

JA and AT: Conceptualization, Investigation, Formal analysis, Visualization, Writing – original draft, Writing – review & editing. BA: Writing ‐ original draft, Writing ‐ review & editing. GMG: Conceptualization, Writing – original draft, Writing – review & editing. QL and RC: Conceptualization, Supervision, Funding acquisition, Resources, Project administration. All authors contributed significantly to the work and approved the final version for submission.

## Supporting information


**Figure S1.** Boxplots accompanied by violins showing the weight of alate and apterous *Myzus persicae* before (pre‐predation) and after (post‐predation) exposure to a single female (A, B) or male (C, D) *Harmonia axyridis* for 24 h. Both alate and apterous groups showed significantly lower weight post‐predation compared to pre‐predation (*** = *P* < 0.001). Biomass data are presented as uncorrected values (natural weight gain of survivors was not quantified).

## Data Availability

The data that support the findings of this study are available on request from the corresponding author. The data are not publicly available due to privacy or ethical restrictions.
