# Supplementary material for: Take flight: predation risk induces wing polyphenism and dispersal in the aphid Myzus persicae
Source: Pest Manag Sci. 2026 Jul 21;82(12):11408–15. doi: 10.1002/ps.71141 (PMC13756825; doi:10.1002/ps.71141)
Supplement: Supplementary file 1 [file PS-82-11408-s001.docx]

**Take flight: predation risk induces wing polyphenism and dispersal in the aphid *Myzus persicae***

**Running title:** Predator risk induces wing polyphenism in aphids

Jamin Ali^1,2^†, Adil Tonğa^3^†, Bashiru Adams^4^, Geoff M. Gurr^5^*, Rizhao Chen^1,2^*, Qiyun Li^1,2^

^1^ College of Plant Protection, Jilin Agricultural University, Changchun, 130118, China

^2^ Sanjiang Laboratory, Changchun, 130118, China

^3^ Entomology Department, Diyarbakır Plant Protection Research Institute, 21110, Diyarbakir, Türkiye

^4^ Delta Research and Extension Center, Mississippi State University, Stoneville, MS 38776, USA

^5^ Gulbali Institute, Charles Sturt University, Orange, New South Wales, Australia

^†^The authors equally share first authorship and are entitled to claim it as first authors on their resumes.

**Correspondence:**

*Geoff M. Gurr <https://orcid.org/0000-0001-5008-7966> [ggurr@csu.edu.au](mailto:ggurr@csu.edu.au)

*Rizhao Chen <https://orcid.org/0000-0001-8866-6648> [rizhaochen@jlau.edu.cn](mailto:rihzaochen@jlau.edu.cn)

**Figure S1** Boxplots accompanied by violins showing the weight of alate and apterous Myzus persicae before (pre-predation) and after (post-predation) exposure to a single female (A, B) or male (C, D) Harmonia axyridis for 24 h. Both alate and apterous groups showed significantly lower weight post-predation compared to pre-predation (*** = P < 0.001). Biomass data are presented as uncorrected values (natural weight gain of survivors was not quantified).
